# SOD3 Expression in Tumor Stroma Provides the Tumor Vessel Maturity in Oral Squamous Cell Carcinoma

**DOI:** 10.3390/biomedicines10112729

**Published:** 2022-10-28

**Authors:** May Wathone Oo, Hotaka Kawai, Htoo Shwe Eain, Yamin Soe, Kiyofumi Takabatake, Sho Sanou, Qiusheng Shan, Yasunori Inada, Masae Fujii, Yoko Fukuhara, Ziyi Wang, Shintaro Sukegawa, Mitsuaki Ono, Keisuke Nakano, Hitoshi Nagatsuka

**Affiliations:** 1 Department of Oral Pathology and Medicine, Graduate School of Medicine, Dentistry and Pharmaceutical Sciences, Okayama University, Okayama 700-8525, Japan; 2 Department of Oral and Maxillofacial Reconstructive Surgery, Graduate School of Medicine, Dentistry and Pharmaceutical Sciences, Okayama University, Okayama 700-8525, Japan; 3 Department of Oral and Maxillofacial Surgery, Graduate School of Medicine, Dentistry and Pharmaceutical Sciences, Okayama University, Okayama 700-8525, Japan; 4 Department of Oral Morphology, Graduate School of Medicine, Dentistry and Pharmaceutical Sciences, Okayama University, Okayama 700-8525, Japan; 5 Department of Molecular Biology and Biochemistry, Graduate School of Medicine, Dentistry and Pharmaceutical Sciences, Okayama University, Okayama 700-8558, Japan; 6 Department of Oral and Maxillofacial Surgery, Kagawa Prefectural Central Hospital, Takamatsu 760-0065, Japan

**Keywords:** oral squamous cell carcinoma, tumor microenvironment, tumor stroma, tumor vascularization, extracellular superoxide dismutase (SOD3), vascular endothelial cadherin (Ve-cadherin)

## Abstract

Tumor angiogenesis is one of the hallmarks of solid tumor development. The progressive tumor cells produce the angiogenic factors and promote tumor angiogenesis. However, how the tumor stromal cells influence tumor vascularization is still unclear. In the present study, we evaluated the effects of oral squamous cell carcinoma (OSCC) stromal cells on tumor vascularization. The tumor stromal cells were isolated from two OSCC patients with different subtypes: low invasive verrucous squamous carcinoma (VSCC) and highly invasive squamous cell carcinoma (SCC) and co-xenografted with the human OSCC cell line (HSC-2) on nude mice. In comparison, the CD34+ vessels in HSC-2+VSCC were larger than in HSC-2+SCC. Interestingly, the vessels in the HSC-2+VSCC expressed vascular endothelial cadherin (VE-cadherin), indicating well-formed vascularization. Our microarray data revealed that the expression of extracellular superoxide dismutase, *SOD3* mRNA is higher in VSCC stromal cells than in SCC stromal cells. Moreover, we observed that SOD3 colocalized with VE-cadherin on endothelial cells of low invasive stroma xenograft. These data suggested that SOD3 expression in stromal cells may potentially regulate tumor vascularization in OSCC. Thus, our study suggests the potential interest in SOD3-related vascular integrity for a better OSCC therapeutic strategy.

## 1. Introduction

Most solid tumors contain a more significant proportion of stroma than other tumors. The tumor stroma plays a critical role in characterizing tumors such as tumor progression, invasion, and metastasis [1,2]. In the head and neck region, oral squamous cell carcinoma (OSCC) is a common malignant tumor and is subclassified as exophytic and endophytic OSCC according to clinical invasiveness [3]. The exophytic OSCC presents outward growth with low invasive character and is also defined as verrucous oral cancer (VSCC). On the other hand, the exophytic OSCC shows a highly invasive ability and is also known as conventional oral cancer (SCC). Recently, studies on the impact of stroma on tumor cells and the immune system have been increasing. It has been reported that a higher proportion of stroma in OSCC relates to poor prognosis [4,5]. We also observed that incorporating different stromal cells into oral cancer xenograft models showed different effects on tumor progression, bone invasion, and bone marrow cell recruitment [6,7]. On the other hand, tumor angiogenesis supports tumor development, dissemination, and metastasis [8].

Tumor angiogenesis is one of the hallmarks of solid tumor development and is essential for the continued survival and progression of tumor cells. There is a shred of evidence that angiogenesis supports tumor development beyond a few milliliters [9,10]. The aggressive tumor growth and associated overexpression of pro-angiogenic factors lead to disorganized vascularization resulting in immature, tortuous, and hyperpermeable characteristics. In addition, an imbalance in the oxidative stress and antioxidant system greatly affects the angiogenesis of various pathologies including tumors. Reduction in the extracellular antioxidant enzyme, superoxide dismutase 3 (SOD3), leads to the accumulation of oxidative free radicals resulting in pathological angiogenesis [11]. The adhesion molecules in endothelial cells are essential for maintaining the vascular architecture. Vascular endothelial cadherin (VE-cadherin), also known as CD144, the component of endothelial junction molecules, functions as a key organizer for stable vascular integrity and a controller for vascular permeability [12,13]. In cancer, endothelial cells impair cell-to-cell adhesion, which is essential for vascular homeostasis and integrity [12]. To our knowledge, the influence of tumor stroma on SOD3 function in tumor angiogenesis and vascular integrity has not been studied yet.

The present study aimed to investigate whether the tumor stroma affects tumor vascularization in OSCC. To this end, we performed investigations on the different tumor stroma transplanted OSCC study models. We isolated the stromal cells from two OSCC patients (low invasive verrucous oral cancer: VSCC and high invasive oral cancer: SCC) and transplanted the different stromal cells with the same human oral cancer cell line (HSC-2) to the nude mice. Our data revealed that tumor stroma has the potential to regulate tumor vascularization, and antioxidant enzyme expression in tumor stromal cells may relate to tumor vascular integrity.

## 2. Materials and Methods

### 2.1. Mice and Cells

Female nude mice (BALB/c-nu/nu mice) were purchased from Shimizu Laboratory Suppliers and were housed under pathogen-free conditions. Human OSCC cell line (HSC-2; JCRB0622) was purchased from JRCB Cell Bank and was grown in minimum essential medium-α (α-MEM) (Life Technologies, Carlsbad, CA, USA) with 10% FBS (Life Technologies, Carlsbad, CA, USA) and 1% antibiotic/antimycotic (Life Technologies, Carlsbad, CA, USA) at 37 °C in a humidified atmosphere with 5% CO_2_. As described previously, the stromal cells from VSCC and SCC patients were isolated [2]. The isolated cells were cultured in α-MEM (Life Technologies, Carlsbad, CA, USA) with 10% FBS (Life Technologies, Carlsbad, CA, USA) and 1% antibiotic/antimycotic (Life Technologies, Carlsbad, CA, USA) at 37 °C in a humidified atmosphere with 5% CO_2_.

### 2.2. Tumor/Stroma Co-Xenograft

Tumor transplantation was performed on nude mice. HSC-2 tumor cells were co-transplanted together with isolated stromal cells VSCC or SCC to nude mice and given the name: HSC-2+VSCC and HSC-2+SCC (n = 4 for each). The cells were mixed at a ratio of 1:3 of tumor and stromal cells (1.0 × 10^6^ HSC-2 cells and 3.0 × 10^6^ stromal cells) and subcutaneously transplanted into the head [14]. After 4 weeks, all mice were sacrificed, and the specimens were harvested for analysis.

### 2.3. Tissue Processing for Histological Analysis

For the formalin-fixed paraffin-embedded sections, the harvested tumor tissues were fixed in 4% paraformaldehyde for 12 h, and decalcification was performed in 10% EDTA at 4 °C for 14 days. Then, the tissues were dehydrated and embedded in paraffin. Serial tissue sections (3 µm) were prepared for the tissue study.

### 2.4. Immunohistochemistry

IHC was performed using the antibodies detailed in Table 1. The sections were deparaffinized in xylene for 15 min and rehydrated in graded ethanol solutions. Endogenous peroxidase activity was blocked by incubating the sections in 0.3% H_2_O_2_ in methanol for 30 min. The antigen in the sections was retrieved, and sections were blocked with 10% normal serum for 15 min. Then, the sections were incubated with primary antibodies at 4 °C overnight. Signals enhancement was performed by the avidin-biotin complex method (Vector Lab, Newark, CA, USA). DAB (Histofine DAB substrate) was used for color development and Myer’s hematoxylin for the counterstaining. The staining results were detected with an optical microscope (BX53, Olympus, Tokyo, Japan).

### 2.5. Double-Fluorescent IHC

Double-fluorescent IHC for CD34/VE-cadherin and CD43/SOD3 was performed using the antibodies detailed in Table 1. Following the antigen retrieval, the sections were incubated in Block Ace (DS Pharma Biomedical, Osaka, Japan) for 20 min at RT. The sections were incubated with a secondary antibody (1:200 dilution) for 1 h at RT. The secondary antibodies used are Alexa Flour 568 anti-rabbit IgG (Abcam, Tokyo, Japan) for CD34, Alexa Flour 488 anti-rat IgG (Abcam, Tokyo, Japan) for VE-cadherin, and Alexa Flour 488 anti-mouse IgG (Abcam, Tokyo, Japan) for SOD3. After the reaction, the sections were stained with DAPI. The staining results were observed with a fluorescence microscope (All-in-One BZ ×700, Keyence, Osaka, Japan).

### 2.6. Multicolor Fluorescent IHC

The OPAL 7-color manual IHC kit was applied to explore the four antigens on the same specimen. We performed the staining procedure according to the manufacturer’s instructions (PerkinElmer, Waltham, MA, USA). Briefly, the cycle of the staining procedure was repeated to detect the antigens separately. After antigen retrieval, incubation in a blocking agent for 10 min and incubation with primary antibodies was performed at 4 °C overnight or 37 °C for 2 h. Then, secondary-HRP for 10 min and signal amplification with Opal-fluorophore for 10 min were applied. Before the next target detection, antigen stripping was performed by microwave heating, and the procedure was repeated from the blocking step for the next target. After detecting all the targets, nucleus staining with DAPI was performed. Finally, sections were visualized using a laser confocal microscope (ZEISS LSM 700, Oberkochen, Germany).

### 2.7. Western Blotting

Western blotting was performed as described previously [7]. 5 μg protein mixed with 4× Laemmli sample buffer (Bio-Rad Laboratories, Hercules, CA, USA) was boiled at 95 °C for 5 min. Each protein sample was separated by SDS-PAGE in 4–20% TGX-GEL (Bio-Rad Laboratories) and transferred to polyvinylidene difluoride membranes (PVDF) (Bio-Rad Laboratories). The membranes were blocked in 1% FBS for 1 h with shaking at RT. Afterward, the membrane was incubated with primary antibodies, mouse anti–SOD3 (1:1000, Sc271170, Santa Cruz Biotechnology, Dallas, TX USA) overnight with shaking at 4 °C. Then incubation with horseradish peroxidase–conjugated (HRP-conjugated) secondary antibodies was performed for 1 h with shaking at RT. Washes before and after antibody reactions were completed on a shaker three times within TBS-T for 10 min each at RT. Alternatively, the detection of tubulin (1:1000, T5168, Sigma AldrichX Burlington, MA, USA) was performed. Finally, blots visualization with ECL substrate (Bio-Rad Laboratories) was performed.

### 2.8. Microarray Data Analysis

The microarray analysis was performed on the stromal cells isolated from low-invasive VSCC and high-invasive SCC. The data are available in the NCBI GEO database (GSE164374) (The data are scheduled to be released on 1 January 2023). Differentially expressed genes (DEGs) of stromal cells were compared. A 2 standard deviation (2SD) was considered as the cut-off value. The biological processes of the DEGs were analyzed using DAVID Bioinformatics Resources (6.8), and oxidation-reduction process-related genes were selected. A heatmap presentation of DEGs of SCC and VSCC was generated using Microsoft Excel.

### 2.9. Quantification and Statistical Analysis

We quantified 5 randomly captured images per mouse (×400 magnification, n = 4) of the tumor sections. The area measurement and cell counting were performed using ImageJ (NIH, v1.52a). All statistical analyses were performed using Graphpad Prism 9.1.1. To compare the two groups, Student’s *t*-test, 2-tailed for independent samples with equal variances, was used. Differences were considered significant at *p* < 0.05 and data are presented as the means ± standard deviation (SD).

## 3. Results

### 3.1. Vascular Architecture Is Different between HSC-2+SCC and HSC-2+VSCC Xenografts

**Figure 1:**
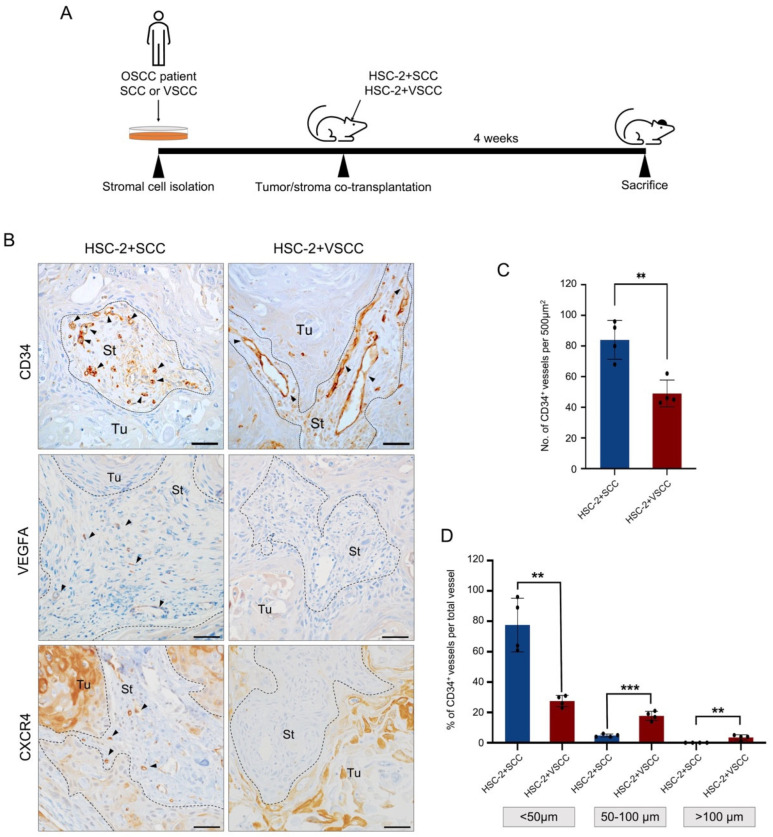
Tumor stroma affects tumor vascularization. (**A**) Illustration of different stroma incorporated oral cancer models establishment. Stromal cells from an oral cancer patient (SCC or VSCC) are isolated. The isolated stromal cells and oral cancer cells (HSC-2) are co-transplanted subcutaneously at the head of the nude mice. After 4 weeks, the mice are sacrificed. (**B**) Representative images of CD34, VEGFA, and CXCR4 IHC staining. Arrows indicate CD34^+^ vessels. Dotted lines represent the boundary of the tumor (Tu) and stroma (St) area. Scale bars: 50 µm. (**C**) The number of CD34^+^ vessels per 500 µm^2^ area. (**D**) The rate of vessels sized <50 µm, 50–100 µm, and >100 µm. All the data are shown as mean ± SD. Statistical analyses were performed using Student’s *t*-test; ** *p* < 0.01, *** *p* < 0.001.

To evaluate the effect of stroma on tumor vascularization, we analyzed the tumor vasculature in two xenografts in which the OSCC tumor cell line (HSC-2) and OSCC patient-derived stromal cells (SCC or VSCC) were co-transplanted (Figure 1A). To identify the vessels, we detected CD34 signals by IHC staining. CD34^+^ cells were in single cells or lumen-shaped vessels (Figure 1B). Then, between two xenografts, we compared the vessel architecture. The quantification was performed by counting CD34^+^ lumens per 500µm^2^ of stromal area, excluding CD34^+^ single cells. Our data showed that the number of vessels was significantly higher in the HSC-2+SCC xenograft than in the HSC-2+VSCC xenograft (Figure 1C). However, the size differed between the two xenografts; while HSC-2+SCC showed small vessels, the HSC-2+VSCC xenograft showed large and long vessels. Therefore, we further analyzed and compared the vascular length in three categories: <50 µm, between 50 µm and 100 µm, and >100 µm. We found that the ratio of blood vessels longer than 50 µm and 100 µm was larger in HSC-2+VSCC xenograft (36%, 7%) compared to HSC-2+SCC xenograft (5%, 0%) (Figure 1D). The blood vessels shorter than <50 µm were more abundant in HSC-2+SCC (95%) compared to HSC-2+VSCC (57%) (Figure 1D). These data indicated that high invasiveness may be associated with a higher number of short and narrow vessels, whereas low invasiveness may be accompanied by a small number of long and large vessels.

Further, we identified the vascularization by detecting the expression of vascular endothelial growth factor A (VEGFA) in the tumor microenvironment. We observed that VEGFA expression in the stromal cells of high invasive stroma xenograft was higher than low invasive stroma xenograft (Figure 1B). In addition, CXCR4-expressed small vessels were largely found in the highly invasive xenograft (Figure 1B). CXCR4 is known to be associated with vascular sprouting [15]. These data indicated that highly invasive stroma promotes vascularization and vessel sprouting.

Thus, different stroma cells may have a different nature to influence the tumor vascularization and highly invasive stroma cells might play a crucial role to promote the tumor vascularization of the OSCC.

### 3.2. HSC-2+VSCC Xenograft Includes VE-Cadherin Expressed Vessels More Than HSC-2+SCC Xenograft

**Figure 2:**
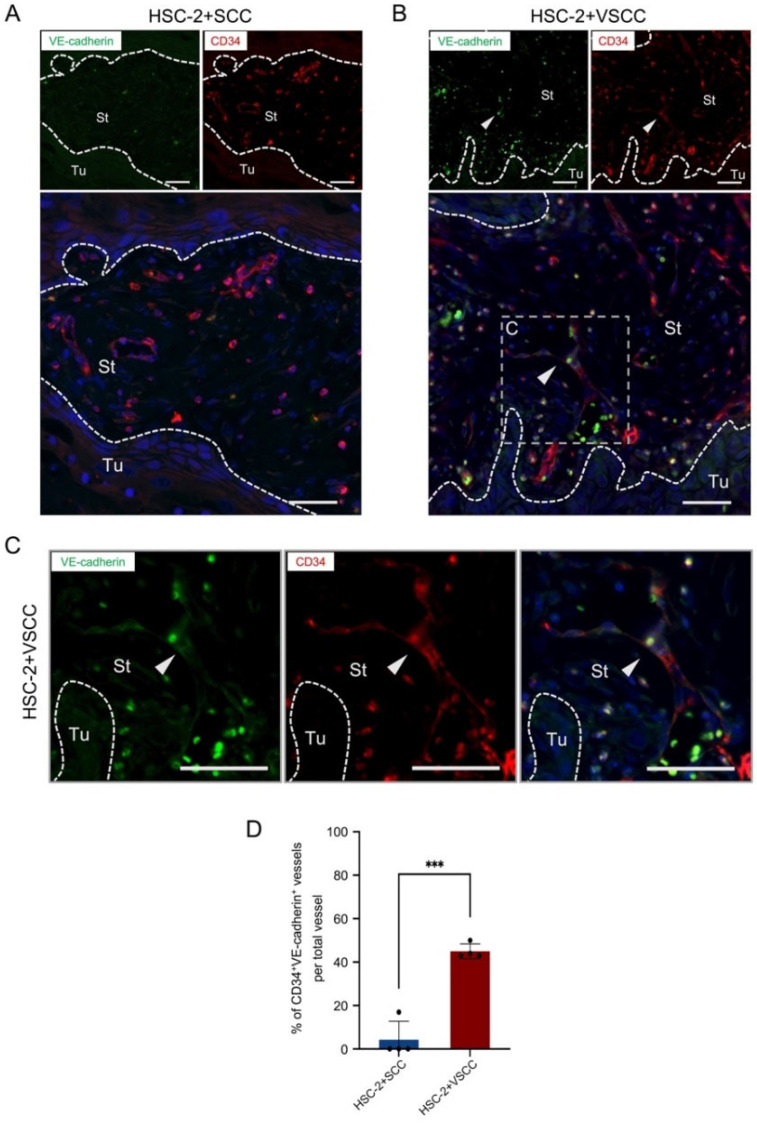
Low invasive VSCC stroma xenograft promotes vascular integrity, while high invasive SCC stroma xenograft shows small vessels. (**A**) Double fluorescent IHC for VE-cadherin (green) and CD34 (red) in HSC-2+SCC xenograft. (**B**) Representative images of double fluorescent IHC for VE-cadherin (green) and CD34 (red) in HSC-2+VSCC xenograft. (**C**) High-magnified images of double fluorescent IHC for VE-cadherin (green) and CD34 (red) in HSC-2+VSCC xenograft. Nuclei are stained with DAPI. Arrowheads indicate double-positive cells. Dotted lines represent the boundary of the tumor (Tu) and stroma (St) area. Scale bars: 50 µm. (**D**) Rate of CD34^+^ VE-cadherin^+^ vessels per total vessels. All the data are shown as mean ± SD. Statistical analyses were performed using Student’s *t*-test; *** *p* < 0.001.

Further, we investigated whether the stroma affects vascular integrity. VE-cadherin is known to express in endothelial cells and support endothelial cell-to-cell adhesion and vascular integrity. Therefore, to measure the maturation of vessels, we detected the VE-cadherin expression in the endothelial cells by double immunofluorescent staining on CD34 and VE-cadherin. Interestingly, VE-cadherin expression was observed in larger vessels suggesting that larger vessels were well-formed and better in integrity (Figure 2A–C). On quantification, HSC-2+VSCC have a higher number of VE-cadherin-expressed vessels (45%) than HSC-2+SCC (4%) (Figure 2D). This result indicated that low invasive stroma: VSCC might promote vascular integrity in OSCC.

### 3.3. SOD3 Expressed Endothelial Cells Composed of the Vessels in HSC-2+VSCC Xenograft

To evaluate whether the antioxidant enzyme is involved in the different tumor vascularization of high- and low-invasive OSCC, we assessed the SOD3 expression in the two xenografts.

**Figure 3:**
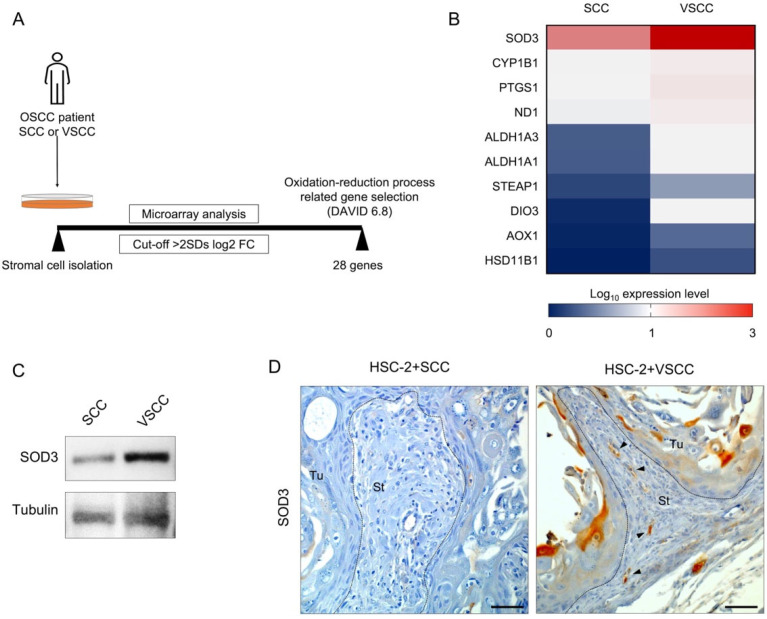
SOD3 expression is higher in low-invasive VSCC stroma than in high-invasive SCC stroma. (**A**) Illustration of oxidation-reduction process-related genes screening. We analyzed the differentially expressed genes (DEGs) of low-invasive VSCC and high-invasive SCC with more than 2 times the standard deviation of fold change cut-off. We selected oxidation-reduction process-related genes by DAVID Bioinformatics Resources 6.8. (**B**) Heatmap presentation of the expression level of oxidation-reduction related genes in SCC stromal cells and VSCC stromal cells. (**C**) Detection of SOD3 in high invasive stromal cells and low invasive stromal cells by western blotting. (**D**) Representative images of SOD3 IHC staining. Arrows indicate SOD3^+^ cells. Dotted lines represent the boundary of the tumor (Tu) and stroma (St) area. Scale bars: 50 µm.

First, we performed the microarray analysis on isolated OSCC patient-derived stromal cells, SCC, and VSCC. In the microarray data analysis, we set the cut-off value of DEGs selection at more than 2SDs log2 ratio of SCC and VSCC. Then, the DEGs related to the oxidation-reduction process were screened using the DAVID 6.8. As data, 28 DEGs involved in the oxidation-reduction process were observed (Figure 3A). Among 28 genes, 10 genes (SOD3, CYP1B1, PTGS1, ND1, ALDH1A3, ALDH1A1, STEAP1, AKR1C8P, SNCA, KDSR, DIO3, AOX1, and HSD11B1) are substantially downregulated in SCC stromal cells compared to VSCC thus confirming that high invasive OSCC decreased oxidation-reduction ability (Figure 3B).

Notably, SOD3 expression was higher in VSCC stroma cells than in SCC stromal cells (Figure 3B). Western blotting showed higher SOD3 expression in VSCC stromal cells than in SCC stromal cells (Figure 3C, a full image of western blotting is provided as Supplementary Figure S1). Further, we confirmed the SOD3 expression in the stroma area of xenografts by SOD3-IHC staining. Like microarray data, SOD3 expression was higher in the stromal area of the HSC-2+VSCC tumor than that in the HSC-2+SCC tumor. Notably, the SOD3 expression localized in the vessel-like lumens or split-like structures, and the staining character was similar to the CD34 staining character (Figure 3D).

**Figure 4:**
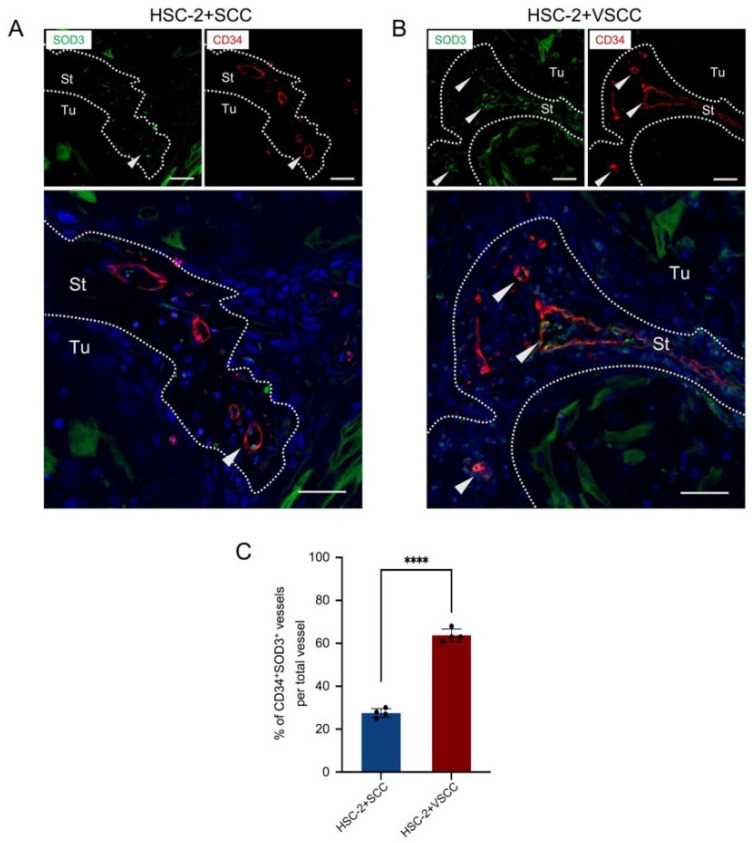
SOD3^+^ endothelial cells form the large vessels in low-invasive VSCC stroma xenograft. (**A**) Representative images of double fluorescent IHC for SOD3 (green) and CD34 (red) in HSC-2+SCC xenograft. (**B**) Representative images of double fluorescent IHC for SOD3 (green) and CD34 (red) in HSC-2+VSCC xenograft. Nuclei are stained with DAPI. Arrowheads indicate double-positive cells. Dotted lines represent the boundary of the tumor (Tu) and stroma (St) area. Scale bars: 50 µm. (**C**) Rate of CD34^+^SOD3^+^ vessels per total vessel. All the data are shown as mean ± SD. Statistical analyses were performed using Student’s *t*-test; **** *p* < 0.0001.

Therefore, double immunofluorescent staining was performed to confirm whether the vessels express SOD3. Our data revealed that SOD3 was expressed in the CD34^+^ endothelial cells of the xenografts (Figure 4A,B). However, the HSC-2+VSCC xenograft showed the significant higher number of SOD3^+^ vessels (64%) than the HSC-2+SCC xenograft (27%) (Figure 4C).

These data indicated that tumor stroma might have the potential to be involved in the correlation of SOD3 and tumor vascularization.

### 3.4. VE-Cadherin Expressed Vessels Show the Association with SOD3 Expression in Endothelial Cells

**Figure 5:**
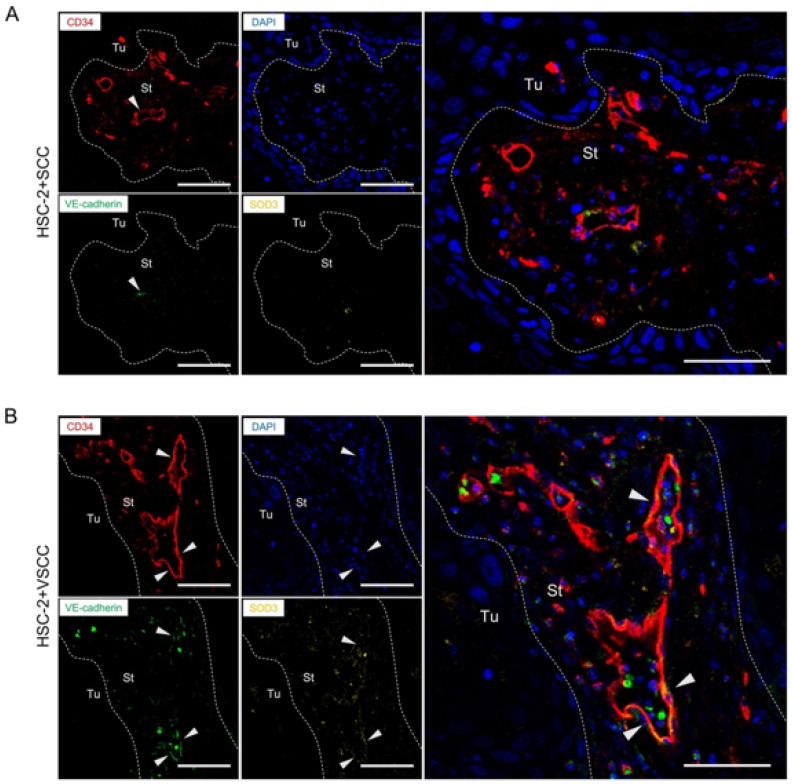
SOD3 expression in stromal cells correlates with vascular integrity (**A**) Representative images of multicolor IHC detection on CD34 (red), VE-cadherin (green), and SOD3 (yellow) in HSC-2+SCC xenograft. (**B**) Representative images of multicolor IHC detection on CD34 (red), VE-cadherin (green), and SOD3 (yellow) in HSC-2+VSCC xenograft. Arrowheads indicate the merged cells. Dotted lines represent the boundary of the tumor (Tu) and stroma (St) area. Scale bars: 50 µm.

Next, to confirm whether SOD3 expression is correlated with vascular maturation, we performed the multicolor visualization of IHC detection on CD34, SOD3, and VE-cadherin on the tumor tissue specimens of the two xenografts. Notably, the larger vessels were composed of SOD3 and VE-cadherin double-positive endothelial cells, while the small vessels were not colocalized in SOD3 and VE-cadherin (Figure 5A,B). HSC-2+VSCC showed the SOD3^+^VE-cadherin^+^ vessels formation but the HSC-2+SCC xenograft did not. As mentioned above, the larger vessels were mainly observed in HSC-2+VSCC, and HSC-2+SCC showed smaller vessel formation. These data indicated that expression of SOD3 in tumor stroma might have the potential to regulate tumor vascularization and improve vascular integrity.

## 4. Discussion

Most solid tumors acquire the nutrients and oxygen for their sustainable progression by sprouting angiogenesis. In cancer, the established tumor vascularization is aberrant, such as abnormalities in architecture, integrity, maturity, and defective vascular functionality [16]. The highly progressive tumors positively correlate with tumor angiogenesis. In this study, we investigated the effect of different stromal cells on tumor vascularization. Accordingly, highly-invasive stroma-transplanted OSCC models showed higher VEGFA expression and higher vascular formation than low-invasive stroma-transplanted OSCC models. CXCR4 is expressed in endothelial cells and promotes angiogenesis. Recent data showed that CXCR4 expression in tumor vessels is associated with poor prognosis in cancers such as oral and liver cancer [15,17]. We observed that the high-invasive stroma xenograft promotes CXCR4 expression in the vessel, but the low-invasive stroma did not. Notably, we used the same oral cancer cell line (HSC-2) for both xenografts, although different isolated stromal cells (SCC or VSCC) were co-transplanted. These findings indicate that tumor stroma may potentially influence tumor vascularization.

The vascularization process is the hierarchical process that involves endothelial cell proliferation, angiogenic sprouting, and maturation. VE-cadherin is the major adhesion molecule of endothelial cells, and VE-cadherin expression is an essential step for vascular integrity and permeability [18]. The deletion of VE-cadherin in the embryo produces impaired vascularization and vascular structures [19,20]. We observed that VE-cadherin expression on tumor vessels differed based on the tumor stromal cells. Low-invasive stromal cells xenograft showed a higher number of larger vessels that expressed VE-cadherin than the high-invasive stromal cells xenograft. Therefore, low-invasive tumor stroma may have the potential to promote tumor integrity rather than increase vascular formation.

There is a shred of evidence and therapeutic strategies to inhibit tumor vessel formation and cut off the nutrients required for tumor progression in many malignant tumors [17,21]. On the other hand, a proper functional vascular formation is necessary for the effectiveness of drug delivery to the tumor microenvironment. Therefore, recent studies are also focusing on the normalization of tumor vessels in the treatment of malignant tumors [22,23,24]. Tumor vessels are known to have abnormal structure and function. Vascular abnormalities in cancer are related to the oxidative stress condition where reactive oxygen species are aberrantly accumulated, and oxidative defenses are poor [25,26,27]. Reactive oxygen species in perivascular areas affect the structure and the cellular components of the endothelium [28]. SOD3 catalyzes the dismutation of superoxide anion (O_2_^−^) into molecular oxygen (O_2_) and hydrogen peroxide (H_2_O_2_) and maintains the oxidative stress condition and reduces the oxidative damage in tissue.

In the tumor microenvironment, antioxidant enzymes such as SOD3 are usually repressed. A low expression of SOD3 is associated with cancer incidence and poor prognosis in cancers such as lung cancer, breast cancer, pancreatic cancer, and prostate cancer [29,30,31,32]. On the other hand, SOD3 highly expressed mesenchymal stem cells showed an immunomodulatory function and potential therapeutic effects in diseases including cancers [33]. In contrast, in oral squamous cell carcinoma (OSCC), a tumor with SOD3 highly expressed cancer cells is related to higher lymph node metastasis than a tumor without SOD3 expression [34]. To our knowledge, the expression of SOD3 in OSCC stromal cells has not been studied yet. We observed that SOD3 expression in the tumor stromal cells was different among the subtypes of OSCC, although the cancer area showed no expression. On Microarray data analysis, SOD3 expression is higher in low-invasive stromal cells than in -high-invasive stromal cells. Consistent with this, tumor tissue of the HSC-2+VSCC xenograft showed a higher expression of SOD3 than the HSC-2+SCC xenograft. These findings suggested that SOD3 is an important factor to pay attention to in the tumor microenvironment. The expression level and location might be the key factors to consider for prognosis prediction and therapeutic strategies.

For many years, it has been known that under the oxidative stress condition, tumors induce their neovascularization by producing angiogenic factors and newly formed tumor vessels are disorganized [35,36,37]. Decreased vascular integrity impairs the blood flow and increases the permeability resulting in increased tumor dissemination and metastasis and limitation of anticancer therapeutic agents [38,39]. Restoration of antioxidant, SOD3, provides the normalization of tumor vessels and improves the delivery of chemotherapeutics into the tumor [40]. SOD3 expression in endothelial cells enhances vasorelaxation, regulates VE-cadherin expression, and enhances the tumor response to chemotherapy [40,41,42]. In the present study, SOD3-expressed vessels were found mainly in the HSC-2+VSCC xenograft. Moreover, the endothelial adhesion molecule, VE-cadherin, is expressed in SOD3-expressed endothelial cells. Taken together, the SOD3 expression in the stromal cells may have the potential to maintain tumor vessel normalization.

## 5. Conclusions

Highly-invasive OSCC had better vascularization than less-invasive OSCC. However, less-invasive OSCC stroma showed higher SOD3 expression than highly-invasive stroma, which may further maintain vascular integrity. Thus, SOD3 might be the potential factor for regulating tumor vascularization in OSCC therapeutic strategy. Since our study is based on nude mice, the study on the relationship between the tumor microenvironment and the immune system is limited. For more knowledge to understand how stromal cells regulate SOD3 expression and tumor vascularization, further studies are needed to investigate.

## Figures and Tables

**Table 1:** Antibodies used in immunohistochemistry.

Primary Antibody	Immunized Animal	Antigen Retrieval	Dilution	Supplier
CD34	Rabbit	Microwave heating in 0.01 mol/L citrate buffer (pH 6.0) at 100 °C for 1 min	1:500	ab81289 ^1^
VEGFA	Rabbit	Microwave heating in 0.01 mol/L citrate buffer (pH 6.0) at 100 °C for 1 min	1:200	ab183100 ^1^
CXCR4	Rabbit	Microwave heating in 0.01 mol/L citrate buffer (pH 6.0) at 100 °C for 1 min	1:300	ab124824 ^1^
VE-cadherin	Rat	Microwave heating in 0.01 mol/L citrate buffer (pH 6.0) at 100 °C for 1 min	1:50	ab282277 ^1^
SOD3	Mouse	Microwave heating in 0.01 mol/L citrate buffer (pH 6.0) at 100 °C for 1 min	1:50	Sc271170 ^2^

^1^ Abcam (Tokyo, Japan), ^2^ Santa Cruz Biotechnology, INC (Dallas, TX, USA).

## Data Availability

The data are available in NCBI GEO (GSE164374) (https://www.ncbi.nlm.nih.gov/geo/query/acc.cgi?&acc=GSE164374, accessed on 10 November 2021). (The data is currently private and is scheduled to be released on 1 January 2024).

## Supplementary Materials

The following supporting information can be downloaded at: https://www.mdpi.com/article/10.3390/biomedicines10112729/s1, Figure S1. Western blotting showing SOD3 and Tubulin in SCC and VSCC stromal cells, supporting Figure 3C. Full images of the western blotting. 20 ug protein sample was applied per each lane. All antigen-antibody reactions were performed using the same membrane.
