# Supplementary material for: SOD3 Expression in Tumor Stroma Provides the Tumor Vessel Maturity in Oral Squamous Cell Carcinoma
Source: Biomedicines. 2022 Oct 28;10(11):2729. doi: 10.3390/biomedicines10112729 (PMC9687713; doi:10.3390/biomedicines10112729)
Supplement: Supplementary file 1 [file biomedicines-10-02729-s001.zip › biomedicines-1947285-supplementary.pdf]

SOD3 expression in tumor stroma provides the tumor vessel maturity in oral squamous cell carcinoma

Supplementary data

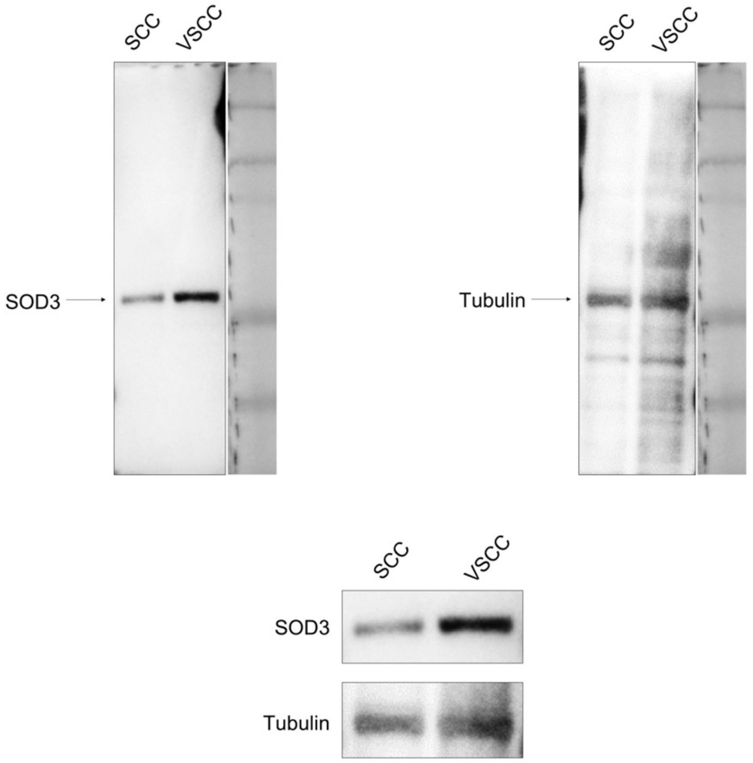

Supplementary Figure S1. Western blotting showing SOD3 and Tubulin in SCC and VSCC stromal cells, supporting Figure 3C. Full images of the western blotting. 20 ug protein sample was applied per each lane. All antigen-antibody reactions were performed using the same membrane.
